# Global health without sexual and reproductive health and rights? Analysis of United Nations documents and country statements, 2014–2019

**DOI:** 10.1136/bmjgh-2020-004659

**Published:** 2021-03-28

**Authors:** Lynda Gilby, Meri Koivusalo, Salla Atkins

**Affiliations:** 1 Health Sciences, Faculty of Social Sciences, Tampere University, Tampere, Finland; 2 New Social Research and Health Sciences, Faculty of Social Sciences, Tampere University, Tampere, Finland; 3 Department of Global Public Health, Karolinska Institutet, Stockholm, Sweden

**Keywords:** health policy, public health, qualitative study

## Abstract

**Introduction:**

The initial International Conference on Population and Development in 1994 contains the first reference to sexual and reproductive health and reproductive rights (SRHR). It has been considered agreed language on SRHR in future United Nations (UN) documents. However, opposition to SRHR in global forums has increased, including in conjunction with an increase in religious, far-right populist politics. This study provides an empirical analysis of UN documents to discover whether opposition to SRHR has resulted in changes in the language on SRHR between and what these changes are.

**Methods:**

This is a qualitative policy analysis in which 14 UN resolutions, 6 outcome documents from the Commission on the Status of Women (CSW) and 522 country and group statements and 5 outcome reports from the Commission on Population and Development were collected from the organisations websites from 2014 to 2019. Framework analysis was used. The text from documents was charted and indexed and themes developed from these.

**Results:**

The results demonstrated a disappearance of the language on abortion in the CSW outcome documents from 2017 and a change in the language on comprehensive sexuality education in the CSW as well as the UN General Assembly resolutions from 2018. This change included a removal of ‘sexuality’ to an increased emphasis on the role of families. Furthermore, documents showed an inability of some states to accept any mention of sexual and reproductive health at all, expanding from the usual contestations over abortion.

**Conclusion:**

Our findings suggest that the global shift in politics and anti-SRHR actors at UN negotiations and conferences have removed previously agreed on language on SRHR from future UN resolutions and outcome documents. This is a concern for the global realisation of SRHR.

**Key questions**

**What is already known?**

- There is a long-standing opposition to sexual and reproductive health and reproductive rights (SRHR) at the United Nations (UN).
- Comprehensive sexuality education, abortion and access to modern contraceptives are contested issues.

**What are the new findings?**

- Contestations expanded to include the inability to accept any reference to SRHR, which created a stalemate in sessions, such as the Commission on Population and Development, leading to an inability to reach an agreement.
- SRHR language differs across UN bodies.
- Opposition to SRHR was proven to be successful in removing previously agreed language on SRHR and replacing it with language which places an emphasis on the role of families.
- The emergence of family-based language was replicated across UN documents, moving from the Commission on the Status of Women outcome document and entering the General Assembly Resolutions.
- There is a risk that this new conservative, watered down language, could become a ‘new normal’, replacing previously agreed SRHR language.

**What do the new findings imply?**

- There needs to be a continued monitoring of negotiations where discussions which include SRHR take place to analyse the efforts to change and remove SRHR language.
- Alliances to defend SRHR must remain strong in their commitment to not allow the change or omission of SRHR from agreements.
- Efforts to defend SRHR must also focus on the international SRHR agenda setting and to make sure that SRHR is realised at the national levels.

## Introduction

International population policies from the 1960s mainly focused on population control, where women’s reproduction was discussed in terms of population targets.^1 2^ These policies targeted women’s fertility, rather than individual autonomy.^2^ In 1984, then United States (US) President Ronald Reagan announced the first Mexico City Policy, also known as the Global Gag Rule, which stated that US federal funding was not to be used to promote abortion as a method of family planning.^3 4^ This contributed to women’s health activists embarking on a global advocacy movement towards more ‘women-centred population policies’, and the recognition of reproductive rights.^5^

In 1994, the United Nations (UN) coordinated the first International Conference on Population and Development (ICPD) which produced the Programme of Action.^6^ This was adopted by 197 governments^7^ and formally recognised the right to sexual and reproductive health (SRH) free from coercion, discrimination and violence.^6^ The ICPD Programme of Action also included the formal recognition of reproductive rights, including the right to safe abortion where legal, as well as adolescents’ rights to reproductive health education.^6^ It provided the framework for sexual and reproductive health and reproductive rights (SRHR) to be included in national health policies^7^ and outlined states obligation to provide unhindered access to SRH services.^2^ In 1995, the Fourth World Conference on Women in Beijing produced the Beijing Declaration Platform for Action, which reconfirmed commitments on SRHR.^8^

The ICPD Programme of Action and Beijing Declaration references to SRHR are considered as ‘qualifying language’ in future UN resolutions pertaining to SRHR.^2^ The establishment of the UN Sustainable Development Goals (SDG’s) for the Agenda 2030 marked a development which saw the inclusion of specific targets recognising SRHR in accordance with the ICPD and Beijing Declaration Platform for Action.^2 7^ Despite this, many countries have been slow to recognise SRHR, with an increasing global effort by countervailing forces through lobbying and direct political interference by powerful UN members,^1 9 10^ such as the previous US Trump Administration.^3 11 12^

Long-standing contestations have mainly focused on access to safe abortion, comprehensive sexuality education (CSE) and modern contraceptives.^10 13 14^ Yet opposition to SRHR, rather than merely abortion, became more explicit with the previous US government under President Donald Trump. This was seen first in the reinstallation and expansion of the Global Gag Rule from previous Republican Administrations, which went further by preventing providers who receive any US Global Health aid from providing abortion services and also from providing any information or referrals to other providers which offer the service,^3^ and were part of the broader politics of silencing on SRHR by the Trump Administration. This was also seen when the Trump Administration used their position as a permanent member of the UN Security Council to threaten to veto a resolution on sexual violence in conflict if it mentioned SRH, which was then subsequently removed.^15^ UN negotiations and high-level meetings also continue to be subject to efforts to replace references to SRHR with conservative language which places an emphasis on traditional, heteronormative family values which exclude individual rights.^2 9 11 12^

State and non-state actors, including religious and conservative non-governmental organisations (NGOs) with links to far-right politicians,^16^ form alliances at the UN to undermine SRHR under the guise of religion, cultural values, traditional values and national sovereignty.^11^ These traditional values relate to family values, which see the exercising of individual rights, particularly sexual and reproductive rights, as a threat to the family.^1 2 9 11 12^ The rise in right-wing political parties globally has also seen a resurgence in religious fundamentalism, where resulting alliances at the UN negotiations have been formed as part of an overall backlash on what is referred to in the literature as ‘gender ideology’,^2 11 17^ a term originally devised by the Catholic church in opposition to the recognition of SRHR in the ICPD and continues to be used by opponents of SRHR.^18^ Side events at the UN provide an opportunity for lobbying activities by both pro-choice and pro-life groups alike.^1 5 19^ Lobbying tactics of anti-choice groups have included forming NGOs and seeking UN consultative status, which provides them access to the UN Economic and Social Council and their subsidiary bodies including the Commission on the Status of Women (CSW) and the Commission on Population and Development (CPD),^9^ in order to influence the SRHR agenda. NGOs with consultative status at the UN have already formed coalitions to promote an anti-abortion, anti-contraception and abstinence only education agenda.^10 13 19^ They lobby conservative governments to appoint delegates which then seek to restrict the language on SRHR.^9 19^ While these lobbying activities have been documented in the literature, to our knowledge, its effect on references to SRHR in a broad range of international documents and how these may have changed over time has not been studied.

Under the political mobilising structures, cross border support between conservative anti-SRHR groups in the US and in the European Union (EU) has meant that these groups have been able to build strategies to undermine SRHR both at the regional level as well as at the UN.^1 5 9 10 18^ The EU and UN have been seen in Poland, Hungary, as well as by right-wing populist parties across Europe, as threatening national identity, sovereignty and family values. Right-wing politicians in Poland have taken a particularly strong stance against sex education and instead placed a focus on the role of motherhood.^20^ Therefore, Poland has diverged from the common EU position which is in support of SRHR.

Anti-SRHR stances at the UN are supported by a coalition which is not reduced to a US Republican Administration alone. In fact, on 22 October 2020, the previous Trump Administration presented the ‘Geneva Consensus Declaration’, an anti-abortion and pro-family document with 31 cosignatories.^21^ Although the opposition to SRHR has been reported in the media^21 22^ and in the literature,^1–3 7 9^ to date, the effect of these efforts on SRHR language across a broad range of policy decisions, from UN resolutions and outcome documents from UN monitoring bodies for the ICPD and Beijing Declaration, has not yet been analysed. While the opposition to SRHR from a US Republican Administration is not new, this study will instead look at what the calls to replace previously agreed SRHR language are and what this has resulted in in practice on the references to SRHR in the documents. The aim of this study was therefore to determine whether the language related to SRHR changed in the UN documents between 2014 and 2019, and what those changes are. Because international guidelines on SRHR can shape policies at the national level, it is crucial to see whether the opposition efforts at the UN has influenced language on SRHR in UN documents.

## Methods

### Study design

This study is a qualitative policy analysis^23^ of publicly available UN resolutions, outcome documents from the CSW, and country statements and outcome reports from the CPD between 2014 and 2019. These years were selected due to the change in influential governments which oppose SRHR during this time and the intensified efforts to roll back SRHR and look at whether this impacted the references to SRHR language in UN documents. Qualitative framework analysis was used as it is widely used in applied policy research^23^ making it particularly suitable for assessing whether there are changes to the language on SRHR in the documents. We did not include 2020 in the analysis due to the COVID-19 pandemic which meant that a political declaration was submitted for the CSW and the CPD had no country statements to analyse. Patients or the public were not involved in the design, or conduct, or reporting, or dissemination plans of our research.

### Data collection

A review of the historical and institutional setting was performed to provide the background on SRHR global policy making and to define the initial SRHR concepts in the ICPD and Beijing Declaration documents. This initial literature search demonstrated the substantial influence of the US on the international SRHR agenda setting and the attempts to expand the anti-SRHR coalition,^3 4 15^ therefore, documents were chosen from 2014 to 2019 to see how the position on SRHR changed between the Democratic Obama Administration to the Republican Trump Administration and how this shaped the debates during the negotiations.

The outcome documents were obtained from the CSW as the CSW monitors and reviews the progress of the implementation of the 1995 Beijing Declaration Platform for Action. The CPD tracks the progress of the 1994 ICPD Programme of Action, so we therefore included the yearly outcome report as well as country and group statements as these demonstrate the position of countries and groups on SRHR, what the arguments against SRHR are and to allow for a further mapping of actors. All country and group statements were included from 2015 to 2019. There were no country statements available in 2014. UN General Assembly resolutions were chosen where SRHR is typically mentioned, which includes resolutions on violence against women and girls. Resolutions from the Human Rights Council (HRC) were included to consider whether the absence of state actors which oppose SRHR may therefore not result in the same changes in the language on SRHR. The resolutions were sourced from the UN digital library at https://digitallibrary.un.org, where a full document text search on SRHR can be applied. The CSW outcome documents are available publicly online from https://www.unwomen.org/en/csw and the CPD statements between 2015 and 2018 from https://www.un.org/development/desa/pd/content/CPD and in 2019 onwards from https://www.un.org/en/development/desa/population/commission/sessions/index.asp. Country and group statements that were not available in English were translated by native speakers in French, Spanish, Arabic, Russian and Chinese. Table 1 provides the list of documents which were obtained for this study. In total, 547 documents were analysed.

**Table 1:** List of documents

Location	Document
Commission on the Status of Women	63rd Session 2019 62nd Session 2018 61st Session 2017 60th Session 2016 59th Session 2015 58th Session 2014
UN General Assembly Resolutions	A/RES/71/170 A/RES/73/148 A/RES/69/147 A/RES/71/170 A/RES/70/137 A/RES/72/146
Human Rights Council Resolutions	A/HRC/RES/29/14 A/HRC/RES/32/4 A/HRC/RES/35/10 A/HRC/38/L.6 A/HRC/RES/29/14 A/HRC/RES/32/4 A/HRC/RES/35/18 A/HRC/RES/39/10
International Conference on Population Development	Programme of Action 1994
Fourth World Conference on Women	Beijing Declaration Platform for Action 1995
Commission on Population Development	2015 Country and group statements 2015 Outcome report 2016 Country and group statements 2016 Outcome report 2017 Country and group statements 2017 Outcome report 2018 Country and group statements 2018 Outcome report 2019 Country and group statements 2019 Outcome report

### Analysis

Each document identified through the search was analysed using framework analysis.^23^ Each category of documentation (eg, CSW outcome documents, CPD statements, resolutions) was read and re-read to identify themes, such as CSE, abortion and access to modern contraceptives, from which the thematic framework was generated. Each document was then indexed and charted onto the relevant thematic framework, mapping the content according to emerging themes from the document category and the year in which the theme emerged, enabling a year-on-year analysis. While charting, new themes emerging in different document categories were included onto the framework of that document category. Examples of emerging themes include ‘family values’, ‘religious’ and ‘cultural context’. The results of this process form the results of this paper, showing the timing, countries and groupings making strong statements on these issues. Examples from the documents are used as quotations or boxes to exemplify content.

## Results

During our analysis, we found the major contested areas to be abortion, CSE and references to ‘sexual and reproductive health’ and ‘sexual and reproductive health and reproductive rights’. We describe first in table 2 how the references to CSE change and how references to abortion disappear from the CSW outcome documents. Following that, in table 3, we demonstrate how a new reference to comprehensive education, which omits ‘sexuality’ and places an emphasis on the role of families, has appeared in the CSW and General Assembly resolutions. Finally, through the CPD statements, we demonstrate the attempt to replace SRHR with family-based language, and the move from the US Trump Administration to refuse to accept any mention of SRHR.

**Table 2:** Commission on the Status of Women outcome documents

Year and page number	Comprehensive sexuality education	Access to modern contraceptives	Access to safe abortion where legal	Preventing adolescent pregnancy
2014, p. 11	‘comprehensive sexual and reproductive health-care services, commodities, information and education’^50^	‘including, inter alia, safe and effective methods of modern contraception, emergency contraception’^50^	‘safe abortion where such services are permitted by national law’^50^	‘prevention programmes for adolescent pregnancy’^50^
2015	Political declaration adopted	Political declaration adopted	Political declaration adopted	Political declaration adopted
2016, p. 8	‘comprehensive sexual and reproductive health-care services, commodities, information and education’^51^	‘including, inter alia, safe and effective methods of modern contraception, emergency contraception’^51^	‘safe abortion where such services are permitted by national law’^51^	‘prevention programmes for adolescent pregnancy’^51^
2017, p. 11	‘universal access to sexual and reproductive health-care services, including for family planning, information and education’^52^	‘universal access to sexual and reproductive health-care services, including for family planning’^52^	X	X
2018, p. 15	Introduction of the ‘double-parent’ paragraph See Panel 1	‘universal access to sexual and reproductive health-care services, including for family planning’^53^	X	X
2019, p. 18	Continuation of the ‘double-parent’ paragraph	‘universal access to sexual and reproductive health-care services, including for family planning’^54^	X	X

2015 marked 20 years since the Beijing Declaration and as such a political declaration was adopted, rather than an outcome document. X indicates a disappearance in the language.

**Table 3:** Comprehensive sexuality education

Year	Human Rights Council	General Assembly	Commission on the Status of Women
2014	–	A/RES/69/147, p. 11 ‘comprehensive sexual and reproductive health-care services, commodities, information and education’^55^	‘comprehensive sexual and reproductive health-care services, commodities, information and education’,^50^ p. 11
2015	A/HRC/RES/29/14, p. 4 ‘access to quality education, including comprehensive sexuality education’^56^	A/RES/70/137, p. 15 ‘comprehensive evidence-based education on human sexuality’^57^	Political declaration adopted
2016	A/HRC/RES/32/4, p. 3 ‘enhance women’s sexual and reproductive health as well as education, providing age appropriate, sexual health information, education’^58^	A/RES/71/170, p. 9 ‘comprehensive education information on sexual and reproductive health, in full partnership with young people, parents, legal guardians, caregivers, educators and health-care providers’^59^	Same paragraph as 2014
2017	A/HRC/RES/35/10, p. 5 ‘comprehensive sexuality education, based on full and accurate information, for all adolescents and youth, in a manner consistent with their evolving capacities, with appropriate direction and guidance from parents and legal guardians’^60^	A/RES/72/146, p. 4 ‘comprehensive education, relevant to cultural contexts, that provides adolescent girls and boys and young women and men, in and out of school, consistent with their evolving capacities, with information on sexual and reproductive health’ ‘in full partnership with young persons, parents, legal guardians, caregivers’^61^	‘universal access to sexual and reproductive health-care services, including for family planning, information and education’,^52^ p. 11
2018	A/HRC/38/L.6, p. 5 Same paragraph as 2017	A/RES/73/148, p. 5 Double-parent paragraph	Double-parent paragraph
2019	–	–	Double-parent paragraph

A ‘-‘ indicates no SRHR pertaining resolution available.

### Commission on the Status of Women

In table 2, we can see that any mention of abortion has completely dropped out of the CSW from 2017 and has not reappeared since then. This includes sovereignty clauses present in both the ICPD and Beijing Declaration that state that ‘*In circumstances where abortion is not against the law, such abortion should be safe. In all cases, women should have access to quality services for the management of complications arising from abortion. Post-abortion counselling, education and family-planning services should be offered promptly, which will also help to avoid repeat abortions’.* 6 24 The introduction of the ‘double-parent paragraph’ in box 1, only references ‘age-appropriate comprehensive education’ and twice mentions the role of parents and legal guardians. Furthermore, ‘prevention programmes for adolescent pregnancy’ has disappeared and been replaced with education to ‘enable to protect themselves from HIV infection and other risks’. Access to ‘modern contraception’ and ‘emergency contraception’ disappeared and was replaced with ‘family planning’.

Box 1 **Commission on the Status of Women 2019 ‘double-parent’ paragraph**

‘Develop policies and programmes with the support, where appropriate, of international organizations, civil society and non-governmental organizations, giving priority to formal, informal and non-formal education programmes, including scientifically accurate and age-appropriate comprehensive education that is relevant to cultural contexts, that provides adolescent girls and boys and young women and men in and out of school, consistent with their evolving capacities, and with appropriate direction and guidance from parents and legal guardians, with the best interests of the child as their basic concern, information on sexual and reproductive health and HIV prevention, gender equality and women’s empowerment, human rights, physical, psychological and pubertal development and power in relationships between women and men, to enable them to build self-esteem and foster informed decision-making, communication and risk-reduction skills and to develop respectful relationships, in full partnership with young persons, parents, legal guardians, caregivers, educators and health-care providers, in order to, inter alia, enable them to protect themselves from HIV infection and other risks.’^54^ p. 18

In table 3, we see that the language relating to CSE differs across the CSW, the General Assembly and the HRC, indicating that the language has not reached a consensus as it changes from year to year. This is until 2018, with the introduction of the double-parent paragraph in box 1. This is the first time in the years analysed that we observed a direct replication of anti-SRHR language, where the double parent paragraph entered both the CSW and the General Assembly. References to the role of parents entered the HRC in 2017 but the ‘double-parent paragraph’ was not included in the HRC resolutions. References to abortion remain present in the HRC resolutions but are only present in General Assembly resolutions on preventing violence against women.

### Commission on Population and Development

Overall, many countries more broadly expressed their commitment to the ICPD Programme of Action, without specifically mentioning SRHR. Common themes arise from statements which do not support SRHR. These include references to ‘family values’ and ‘traditional families’, as well as calls for the respecting of national sovereignty and cultural practices. There are also countries which only referenced ‘reproductive health’ rather than SRH or SRHR. The changing support for SRHR by the US, however, is also included as this was the only country observed which showed such a dramatic regression in support for SRHR which was observed during the Trump Administration.

In 2015, the Country Statement from Nigeria only mentioned ‘maternal health’ and further indicated that ‘sexuality issues’ should be given ‘less mention’.^25^ The outcome reports of the 2015 CPD highlighted that ‘*the issue of sexual and reproductive health and rights was again one of the most contentious’* 26 and can be found in box 2.

Box 2 **2015 CPD Report**

‘The issue of sexual and reproductive health and rights was again one of the most contentious in the Commission on Population and Development, although the importance of access to reproductive health, as well as of reproductive rights, was broadly recognized. For a number of countries, sexual and reproductive health and rights were key to achieve the aims of the Programme of Action of the International Conference on Population and Development, as they were essential for the full realization of all human rights, as well as for ending discrimination and eliminating harmful practices. In line with this, it was also important to promote comprehensive sexuality education. Other countries, however, did not consider sexual and reproductive health and rights an internationally agreed concept. They stressed that they could not accept a resolution that ran counter to their national laws, and reiterated the importance of including references to national sovereignty. They also stressed that sexuality education and related matters should be considered within the national context, including cultural values and religious beliefs. Issues relating to the concept of family and the role of families remained another area of disagreement.’^26^ p. 12

CPD, Commission on Population and Development

There were also countries which consistently only referred to ‘reproductive health’, which included Poland, Kenya, Pakistan, Russia, Qatar, Sudan, India, Iran, Liberia, Malaysia, Maldives, Philippines, Uganda, Kyrgyzstan and Gambia. Some countries also shied away from mentioning access to modern contraception, while others called for their use among married women only. While the EU group statement consistently highlighted their support for SRHR, including CSE, from year to year, Poland would submit a separate country statement which did not mention CSE but instead a teaching programme called ‘*Education for family life’*.^27–30^ As well as only referring to ‘reproductive health’, Poland would also mention ‘*access to methods and means of conscious procreation’*.^27^ Opposition to abortion was expressed yearly by Malta, Nauru, Micronesia, the Holy See and from 2018 the USA, where calls were made for abortion not to be used as a method of family planning. In 2019, Malta specified that SRH should not include abortion, stating that they do ‘*not agree with the interpretation that the right to sexual and reproductive health services includes an intrinsic right to abortion services’*.^31^ Nauru also reaffirmed that they ‘*would like to make clear that we do not view the promotion of abortion as a means of achieving sustainable development’*.^32^

The Holy See was continuously explicitly against SRHR, and instead committed to ‘*address the real needs of mothers and children, especially those unborn’*.^33^ In 2017, The Holy See stated that ‘*While responsible parenthood and sexual behavior are always moral imperatives, the coercive regulation of fertility, especially under the guise of empowerment and rights, undermines individual freedom and responsibility’*.^34^ In 2018, the Holy See again stated their objection to SRHR highlighting reasons of state sovereignty ‘*It is regrettable that this process was derailed because of an inordinate focus on issues related to sexual and reproductive health and reproductive rights and an unwillingness to accept a reference to State sovereignty’*.^35^

References to the ‘traditional family’ was a common occurrence. In 2015, Russia refers to ‘*strengthening the institution of the traditional family’,* 36 and in 2017 Belarus referred to the ‘*strengthening of family values’*.^37^ Belarus would also often refer to raising the ‘prestige’ of motherhood and parenthood. In 2016, Belarus stated that ‘*measures are applied to develop and strengthen the family values and raise the prestige of parenthood’* and that ‘*systematic governmental policy aiming at strengthening and supporting of a family as a basic social institution’*.^38^ In 2019, Belarus stated their priority was for ‘*stimulating the birth rate’* and ‘*increasing the prestige of motherhood’*.^39^ Azerbaijan committed to ‘*Promoting family values and strengthening family institution’*.^40^ In 2019, Russia stated that it ‘*does not bind itself with obligations to introduce the so-called ‘comprehensive sexuality education’ for young people. Parents or guardians should carry the first priority responsibility for sex education for adolescents and young people’*.^41^ Russia also explicitly rejected sexual rights saying that it is ‘*With regret we note that in two of the three reports of the UN Secretary General we see the statements about the so-called ‘sexual rights’ - a concept that is not enshrined in the Cairo Agenda and does not find consensus among member countries’.* 41

Some country and group statements called for the respect of religious and cultural context, such as Qatar and Saudi Arabia. In 2016, The African Group does not mention SRHR but wished ‘*to reaffirm the sovereign right of each Country to implement the recommendations of the Programme of Action or other proposals in the CPD resolutions, consistent with national laws and development priorities, with full respect for the various religious and ethical values and cultural backgrounds of its people, and in conformity with universally recognized international human rights’*.^42^ The Gulf Cooperation Council statement said they ‘*would like to refer particularly to the phrases “early marriage” and “sexual and reproductive rights”. In discussing these subjects, we reaffirm the importance of taking into consideration the national, regional, historical, cultural and religious backgrounds of state’.* 43

The country statements from the US were the only ones identified which changed dramatically over the years analysed. In 2015, the US country statement, under the Obama Administration, referenced their ‘*strong support’* for SRH including ‘*universal access to sexual and reproductive health services’*.^44^ In 2016, still under the Obama Administration, reaffirmed their support for SRHR, including the necessity of adolescent access to CSE. The 2017 country statement when discussing addressing maternal mortality and morbidity does not mention access to SRH, and only mentions ‘*the importance of reproductive health’* thereby specifically excluding sexual health, or any other SRHR reference. The 2017 CPD outcome report highlights the contestations around SRHR stating that ‘*Delegations disagreed about the meaning of these terms and whether their use required qualification’* 45 (see box 3).

Box 3 **2017 CPD Report**

‘Promoting sexual and reproductive health and reproductive rights. Issues surrounding sexual and reproductive health and reproductive rights were again among the most contentious in discussions within the Commission. Delegations disagreed about the meaning of these terms and whether their use required qualification. For example, there was disagreement about whether these terms should be qualified by adding “in accordance with the Programme of Action of the International Conference on Population and Development and the Beijing Platform for Action”. This proposal was supported by some delegations but unacceptable to others, and thus there was no agreement. Regarding sexuality education, the Chair’s text qualified the reference to comprehensive sexuality education by specifying that such education should be “age appropriate”. Delegations still disagreed about the use of the phrase “comprehensive education on human sexuality”. There were calls to delete the word “comprehensive” or the entire phrase. These proposals were unacceptable to some delegations, and thus there was no agreement.’^45^ p. 12

CPD, Commission on Population and Development

In 2018, there was a limited number of statements available, and the outcome report states that the commission did not reach a consensus that year for ‘*reasons relating to sexual and reproductive health and national sovereignty’*.^46^ That year, the US was unable to accept the text due to ‘*unqualified references to sexual and reproductive health’*,^47^ while in contrast, a joint statement from 35 EU and non-EU countries noted ‘*with concern the lack of reference to sexual and reproductive health and rights, which are at the heart of sustainable development*.’^48^ They further wrote that this was the third time ‘*in a matter of a few years only’* that the commission was unable to reach a consensus ‘*despite our joint efforts and constructive engagements of delegations to reach an outcome throughout this week*.’^48^ The 2019 Statement by the US only discusses access to maternal and child health and adds ‘we continue *to insist that references to ‘sexual and reproductive health services’ in the ICPD Programme of Action do not include abortion or the promotion of abortion as a method of family planning’*.^49^ Despite the fact that ‘sexual and reproductive health’ is already agreed language from the ICPD and Beijing documents, it is not clear from the 2018 US Country Statement exactly which references to SRH they are referring to and which they suggest are ‘unqualified’. Alternatively, this could imply that the Trump Administration could not accept any reference to SRH at all.

## Discussion

Our study shows that there have been multiple changes to the language on SRHR in UN outcome documents and resolutions to focus more on families, as well as calls to reject any mention of SRHR at all. Furthermore, references to SRHR fail to reach an agreement across UN bodies.

There has been a complete removal of any mention of abortion from the CSW, and a change in CSE language which places an emphasis on the role of families and removes the word ‘sexuality’ from CSE, which is in line with the findings from the literature.^2 9 10^ However, what our study further highlights is the infiltration of the ‘double parent language’ on CSE which has moved between the CSW and the General Assembly, but not yet the HRC. This demonstrates the ability to shift anti-SRHR, family-based language across UN bodies and into other UN documents.

Abortion has been completely omitted from the CSW, as well as the qualifying language on abortion which included sovereignty clauses on safe abortion where legal, and the provision of services for dealing with unsafe abortion have also disappeared from the CSW, marking a roll back from the ICPD and Beijing Declaration commitments. Abortion has not been omitted from the General Assembly and HRC resolutions at the time of writing. The CPD country statements show a pervasive opposition to the provision of abortion services. This is despite both the ICPD and Beijing documents including sovereignty clauses that state ‘In circumstances where abortion is not against the law, such abortion should be safe’ and also reiterate that ‘In no case should abortion be promoted as a method of family planning’.^7^

Despite the statement from the EU States supporting SRHR to include CSE at the CPD, Poland entered their own statement specifying their support for reproductive health while notably leaving out internationally agreed language on SRH. This offers support to the literature^17 18^ which demonstrates the opposition to SRHR from Poland and shows their divergence with the common EU position at international negotiations.^18^ Instead of access to modern contraceptives, Polish statements talk about ‘means of conscious procreation’, as well as promoting ‘education on family life’ instead of CSE.^27–30^

There are consistent references to family-based language, such as ‘the importance of family values’ and ‘strengthening the family institution’. Furthermore, the CPD statements have shown that opposition had hardened from CSE and abortion to any mention of SRHR at all under the previous US Trump Administration. The language in the CPD country statements differed markedly between the Democratic Obama and the Republican Trump Administrations, and the US statements showed a substantial change on government stances on SRHR which was not evidenced in the other country/group statements. With the Mexico City Policy, SRHR has become a partisan issue where domestic politics on abortion are reflected strongly in US foreign policy.^2–4 7 10 11 19^ As already shown in the threat to veto the UN Security Council resolution on sexual violence in conflict, the move to not accept any reference to SRH or SRHR will have an impact in other discussions which include SRHR,^15^ such as Universal Health Coverage. With the US being the largest donor of global health development aid, it has a substantial influence of the international SRHR agenda.^3^

The removal of abortion from the CSW, the prior existence of the expanded Mexico City Policy, and the objections to SRHR in the CPD country statements demonstrate an attempt to silence any mention of abortion through foreign policy. With international guidelines on SRHR being able to form the framework of policies at the national level, the consequences of this could mean that these services could be left out of national health provisions even in countries where abortion is legal.

## Conclusion

Our study provides empirical evidence on the disappearance of the references to abortion and a changing of the language on CSE from UN resolutions and CSW outcome documents. The inability of states to accept SRHR furthermore created a stalemate in sessions, such as the CPD, which lead to an inability to reach an agreement. Overall, opposition to SRHR has proven to be successful in removing previously agreed language on SRHR and replacing it with language which places an emphasis on the role of families. The emergence of this ‘traditional family-based language’, as seen with the ‘double-parent’ paragraph, also has the ability to be replicated across UN bodies. There is therefore a risk that this new conservative, watered down language, could become a ‘new normal’, replacing previously agreed SRHR language. There is also a risk that SRHR could be completely removed, as seen with the omission in the UN Security Council resolution, in order to pass broader commitments. Even though the new US Biden Administration has rescinded the Mexico City Policy and withdrawn the US signature from the Geneva Consensus Declaration, the coalition-building against SRHR is likely to continue as opposition to SRHR is already on the agenda of several other governments. This will continue to create challenges to defending SRHR in UN negotiations as the ability to change and remove previously agreed SRHR language and replace it with ‘family-based’ language is likely to have a substantial impact in other international, regional and national policy making forums where women’s health, gender equality, and sexual and reproductive rights are discussed. Allies must continue to monitor negotiations where discussions which include SRHR take place to ensure alliances to defend SRHR remain strong in their commitment to not allow the change or omission of SRHR from agreements. Efforts to defend SRHR must also focus on the international SRHR agenda setting and to make sure that SRHR is realised at the national levels. One of the limitations of this study is that it does not provide a more nuanced analysis of the broader political dynamics involved in the opposition to SRHR, which is beyond the scope of this study and is an area which requires further study.

## Data Availability

Data are available in a public, open access repository. Data are available in a public, open access repository from https://digitallibrary.un.org, https://www.unwomen.org/en/csw, https://www.un.org/development/desa/pd/content/CPD, and https://www.un.org/en/development/desa/population/commission/sessions/index.asp.

## References

[1] Eager P . Global population policy: from population control to reproductive rights. New York: Routledge, 2004.

[2] Berro Pizzarossa L . Here to stay: the evolution of sexual and reproductive health and rights in international human rights law. Laws 2018;7:29. 10.3390/laws7030029

[3] Brooks N , Bendavid E , Miller G . Usa aid policy and induced abortion in sub-Saharan Africa: an analysis of the Mexico City policy. Lancet Glob Health 2019;7:e1046–53. 10.1016/S2214-109X(19)30267-0 31257094

[4] Crane BB , Dusenberry J . Power and politics in international funding for reproductive health: the US global Gag rule. Reprod Health Matters 2004;12:128–37. 10.1016/s0968-8080(04)24140-4 15626203

[5] Joachim JM , Setting A . The un, and NGOs gender violence and reproductive rights. Washington, D.C: Georgetown University Press, 2007.

[6] Osotimehin B . Programme of action of the International Conference on population sevelopment. New York, 1999. Available: https://www.unfpa.org/sites/default/files/pub-pdf/programme_of_action_WebENGLISH.pdf

[7] Hadi M . Historical development of the global political agenda around sexual and reproductive health and rights: a literature review. Sex Reprod Healthc 2017;12:64–9. 10.1016/j.srhc.2017.03.005 28477934

[8] Carroll A , Perolini AC . Human rights references to sexual and reproductive health and rights. ILGA Europe, 2007. Available: https://www.ilga-europe.org/sites/default/files/Attachments/webversion.pdf

[9] NORAD . Lobbying for faith and family: a study of religious NGOs at the United nations, 2013. Available: https://www.oursplatform.org/wp-content/uploads/lobbying-for-faith-and-family.pdf

[10] Nowicka W . Sexual and reproductive rights and the human rights agenda: controversial and contested. Reprod Health Matters 2011;19:119–28. 10.1016/S0968-8080(11)38574-6 22118146

[11] Franklin S , Ginsburg F . Reproductive politics in the age of Trump and Brexit. Cultural Anthropology 2019;34:3–9. 10.14506/ca34.1.02

[12] Lemon KD . Sex education in America: Abstaining from comprehensive facts. J Midwifery Womens Health 2019;64:149–53. 10.1111/jmwh.12901 30329214

[13] Alldred P , David ME . Get real about sex the politics and practice of sex education. Maidenhead, Berkshire, England: McGraw-Hill Education, 2007.

[14] Defago P , Angélica M , Faúndes M . Conservative litigation against sexual and reproductive health policies in Argentina conservative litigation against sexual and reproductive health policies in Argentina. Reproductive Health Matters 2014;22:82–90. 25555765 10.1016/S0968-8080(14)44805-5

[15] Gramer R , Lynch C . How a U.N. Bid to prevent sexual violence turned into a Spat over abortion. foreignpolicy.com, 2019. Available: https://foreignpolicy.com/2019/04/23/united-nations-bid-end-sexual-violence-rape-support-survivors-spat-trump-administration-sexual-reproductive-health-dispute-abortion-internal-state-department-cable

[16] Brough M , Snip I , Provost C , et al . Interactive: Explore US Christian right 'dark money' spending globally. opendemocracy.net, 2020. Available: https://www.opendemocracy.net/en/5050/interactive-explore-us-christian-right-dark-money-spending-globally/

[17] Vida B . New waves of anti-sexual and reproductive health and rights strategies in the European Union : the anti-gender discourse in Hungary. Sexual and Reproductive Health Matters 2019;27:13–16. 10.1080/26410397.2019.1610281 PMC7887896 31533587

[18] Hodzic A , Bijelic N . NEO-CONSERVATIVE THREATS TO SEXUAL AND REPRODUCTIVE HEALTH & RIGHTS IN THE EUROPEAN UNION. CESI, 2014. Available: https://www.rosadoc.be/digidocs/dd-000557_2014_neo-conservative_threats_to_srhr_in_eu.pdf

[19] Chamberlain P . UNdoing reproductive freedom: Christian right NGOs target the United nations. political research associates, 2006. Available: http://www.publiceye.org/reproductive_rights/UNdoingReproFreedomSimple.html

[20] Kováts E , Poim M . Gender as symbolic glue, 2015. Available: https://library.fes.de/pdf-files/bueros/budapest/11382.pdf

[21] Borger J . US signs anti-abortion Declaration with group of largely authoritarian governments. The guardian, 2020. Available: https://www.theguardian.com/world/2020/oct/22/us-trump-administration-signs-anti-abortion-declaration?CMP=Share_iOSApp_Other

[22] Sardana N . Prioritizing Gender in Universal Health Coverage. International Women’s Health Coalition, 2019. Available: https://iwhc.org/2019/09/the-inside-story-of-the-uns-political-declaration-on-universal-health-coverage/

[23] Srivastava A , Thomson S . Framework analysis: a qualitative methodology for applied policy research. J Adm Gov 2009;4:72–9.

[24] United Nations . Beijing Declaration and platform for action. New York, 1995. Available: https://www.unwomen.org/-/media/headquarters/attachments/sections/csw/pfa_e_final_web.pdf?la=en&vs=800

[25] Sarki U , Statement by HE . Usman Sarki Nigeria Ambassador/Deputy permanent representative at 48th session of the United nations Commission on population and development. New York, 2015. Available: https://www.un.org/en/development/desa/population/pdf/commission/2015/country/Agendaitem4/Nigeria_Item4

[26] United Nations . Commission on population and development report on the forty-eighth session, 2015. Available: https://www.un.org/en/development/desa/population/commission/pdf/48/N1513807English.pdf

[27] Republic of Poland . Review and appraisal of the programme of action of the International Conference on population and development and its contribution to the follow-up and review of the 2030 agenda for sustainable development. New York, 2019. Available: https://www.un.org/en/development/desa/population/pdf/commission/2019/country/AgendaItem3/poland_en.pdf

[28] Potrykowska A , Ms Sby . Alina Potrykowska Secretary General government population Council of Poland Commission on population and development 48th session. New York, 2015. Available: https://www.un.org/en/development/desa/population/pdf/commission/2015/country/Agendaitem4/Poland_Item4.pdf

[29] Pinkas J , Mr Sby . Jaroslaw Pinkas Secretary of State at the Ministry of Health of the Republic of Poland Commission for Population and Development 49th Session Agenda Iten 4 - General Debate on the National Experience in Population Matters. New York, 2016. Available: https://www.un.org/en/development/desa/population/pdf/commission/2016/country/Agendaitem4/Poland_EN_Item4.pdf

[30] Potrykowska A , Ms Sby . Alina Potrykowska Secretary General of the government population Council of the Republic of Poland Commission for population and development 50th session agenda item 3: general debate: actions for the further implementation of the program. New York, 2017. Available: https://www.un.org/en/development/desa/population/pdf/commission/2017/country/AgendaItem3/poland_en.pdf

[31] Inguanez HE. H.E . Ambassador Carmelo Inguanez Ambassador and permanent representative ofthe Republic of Malta to the United nations. United nations, 2019. Available: https://www.un.org/development/desa/pd/sites/www.un.org.development.desa.pd/files/CPD/52/documents/statements/country/agendaitem3/malta_en.pdf

[32] United Nations . Draft statement Commission on population and development Fifty-Second session 1st to 5th April 2019, 2019. Available: https://www.un.org/development/desa/pd/sites/www.un.org.development.desa.pd/files/CPD/52/documents/statements/country/agendaitem3/nauru_en.pdf

[33] Nuncio A , Statement of HE . Archbishop Bemardito Auza Apostolic Nuncio, permanent observer of the Holy see, 2019. Available: https://www.un.org/development/desa/pd/sites/www.un.org.development.desa.pd/files/CPD/52/documents/statements/country/agendaitem3/holy-see_en.pdf

[34] Intervention of H.E Nuncio A . Archbishop Bernardito Auza Apostolic Nuncio and permanent observer of the Holy see to the United nations, 2017. Available: https://www.un.org/en/development/desa/population/pdf/commission/2017/country/AgendaItem3/holy-see_en.pdf

[35] Nuncio A , Statement of HE . Archbishop Bemardito Auza Apostolic Nuncio and Permanent Observer of the Holy See to the United Nations Fifty-First Session of the Commission on Population and Development Agenda Item 3(b): Sustainable cities, human mobility and internat, 2018. Available: https://www.un.org/en/development/desa/population/pdf/commission/2018/country/AgendaItem3/en_holy-see.pdf

[36] United Nations . Statement from the Russian Federation on the 48th session of the Commission on population and development, 2020. Available: https://www.un.org/en/development/desa/population/pdf/commission/2015/country/Agendaitem4/Russia_Item4.pdf

[37] United Nations . Statement from Belarus on the 50th session of the Commission on population and development, 2017. Available: https://www.un.org/en/development/desa/population/pdf/commission/2017/country/AgendaItem3/belarus_ru.pdf

[38] United Nations . Statement from Belarus on the 49th session of the Commission on population and development, 2016. Available: https://www.un.org/en/development/desa/population/pdf/commission/2016/country/Agendaitem4/Belarus_RU_Item4.pdf

[39] United Nations . Statement from Belarus on the 52nd session on the Commission on population and development, 2019. Available: https://www.un.org/development/desa/pd/sites/www.un.org.development.desa.pd/files/CPD/52/documents/statements/country/agendaitem3/belarus_ru.pdf

[40] United Nations . 50Th session of the Commission on population and development General discussion on item 3 statement by the delegation of Azerbaijan 05 April 2017, 2017. Available: https://www.un.org/en/development/desa/population/pdf/commission/2017/country/AgendaItem3/azerbaijan_en.pdf

[41] United Nations . Statement from the Russian Federation on the 52nd session of the Commission of population and development, 2019. Available: https://www.un.org/development/desa/pd/sites/www.un.org.development.desa.pd/files/CPD/52/documents/statements/country/agendaitem3/russian-federation_ru.pdf

[42] Nduhuura R , Statement by HE , Richard Nduhuura DR . Permanent representative of the mission of Uganda, on behalf of the African group at the 49th session of the Commission on population and development, 2016. Available: https://www.un.org/en/development/desa/population/pdf/commission/2016/country/Agendaitem4/Uganda_OBO_AfricanGroup_EN_Item4.pdf

[43] United Nations . Statement on behalf of the Gulf cooperation Council. New York, 2016. Available: https://www.un.org/en/development/desa/population/pdf/commission/2016/country/AfterClosing/Qatar_EN.pdf

[44] Pollack M . Statement by the United States at the 48th un Commission on population and development. New York, 2015. Available: https://www.un.org/en/development/desa/population/pdf/commission/2015/country/Agendaitem4/United-States_Item4.pdf

[45] ODS - Sédoc . Commission on population and development report on the forty-seventh session, 2017. Available: https://documents-dds-ny.un.org/doc/UNDOC/GEN/N17/114/84/PDF/N1711484.pdf?OpenElement

[46] ODS - Sédoc . Commission on population and development report on the fifty-first session (7 April 2017 and 9–13 April 2018) economic and social Council official records, 2018 supplement No. 5. New York, 2018. Available: https://documents-dds-ny.un.org/doc/UNDOC/GEN/N18/127/89/PDF/N1812789.pdf

[47] United Nations . Commission on population and development 51st session United States closing statement. New York, 2018. Available: https://www.un.org/en/development/desa/population/pdf/commission/2018/country/closing/en_usa.pdf

[48] United Nations . CPD51 joint statement, 2018. Available: https://www.un.org/en/development/desa/population/pdf/commission/2018/country/closing/en_tunisia.pdf

[49] Chalet CN. U.S . Statement: un Commission on population and development, 52nd session. New York, 2019. Available: https://www.un.org/en/development/desa/population/pdf/commission/2019/country/AgendaItem3/usa_en.pdf

[50] UN Women . Challenges and achievements in the implementation of the millennium development goals for women and girls. 2014 Commission on the status of women agreed conclusions. New York, 2014. Available: https://www.unwomen.org/-/media/headquarters/attachments/sections/csw/58/csw58_agreed_conclusions.pdf?la=en&vs=1525

[51] UN women . Women’s Empowerment and the Link to Sustainable Development: 2016 Commission on the Status of Women Agreed Conclusions. New York, 2016. Available: https://www.unwomen.org/-/media/headquarters/attachments/sections/csw/60/csw60agreedconclusionsconclusionsen.pdf?la=en&vs=4409

[52] UN women . Women’s Economic Empowerment in the Changing World of Work. 2017 Commission on the Status of Women Agreed Conclusions. New York, 2017. Available: https://www.unwomen.org/-/media/headquarters/attachments/sections/csw/61/csw-conclusions-61-web.pdf?la=en&vs=5452

[53] UN Women . Challenges and opportunities in achieving gender equality and the Empowerment of rural women and girls. 2018 Commission on the status of women agreed conclusions. New York, 2018. Available: https://www.unwomen.org/-/media/headquarters/attachments/sections/csw/62/csw-conclusions-62-en.pdf?la=en&vs=4713

[54] United Nations . Social protection systems, access to public services and sustainable infrastructure for gender equality and the empowerment of women and girls. agreed conclusions, 2019. Available: https://undocs.org/en/E/CN.6/2019/L.3

[55] United Nations . Resolution adopted by the general assembly on 18 December 2014. 69/147. intensification of efforts to eliminate all forms of violence against women and girls, 2015. Available: https://undocs.org/en/A/RES/69/147

[56] United Nations . Resolution adopted by the human rights Council on 2 July 2015. 29/14. accelerating efforts to eliminate all forms of violence against women: eliminating domestic violence, 2015. Available: https://undocs.org/en/A/HRC/RES/29/14

[57] United Nations . Resolution adopted by the general assembly on 17 December 2015. 70/137. rights of the child, 2016. Available: https://undocs.org/en/A/RES/70/137

[58] United Nations . Resolution adopted by the human rights Council on 30 June 2016 32/4. elimination of discrimination against women, 2016. Available: https://undocs.org/en/A/HRC/RES/32/4

[59] United Nations . Resolution adopted by the general assembly on 19 December 2016. 71/170. intensification of efforts to prevent and eliminate all forms of violence against women and girls: domestic violence, 2017. Available: https://undocs.org/en/A/RES/71/170

[60] United Nations . Resolution adopted by the human rights Council on 22 June 2017. 35/10. accelerating efforts to eliminate violence against women: engaging men and boys in preventing and responding to violence against all women and girls, 2017. Available: https://undocs.org/en/A/HRC/RES/35/10

[61] United Nations . Resolution adopted by the general assembly on 19 December 2017. 72/146. policies and programmes involving youth, 2018. Available: https://undocs.org/en/A/RES/72/146

